# Gastrointestinal Stromal Tumor – An Evolving Concept

**DOI:** 10.3389/fmed.2014.00043

**Published:** 2014-11-11

**Authors:** Luigi Tornillo

**Affiliations:** ^1^Institute of Pathology, University of Basel, Basel, Switzerland

**Keywords:** gastrointestinal stromal tumors, gastrointestinal tract, CKIT, targeted therapy, receptor tyrosine kinase

## Abstract

Gastrointestinal stromal tumors (GISTs) are the most frequent mesenchymal tumors of the gastrointestinal tract. The discovery that these tumors, formerly thought of smooth muscle origin, are indeed better characterized by specific activating mutation in genes coding for the receptor tyrosine kinases (RTKs) CKIT and PDGFRA and that these mutations are strongly predictive for the response to targeted therapy with RTK inhibitors has made GISTs the typical example of the integration of basic molecular knowledge in the daily clinical activity. The information on the mutational status of these tumors is essential to predict (and subsequently to plan) the therapy. As resistant cases are frequently wild type, other possible oncogenic events, defining other “entities,” have been discovered (e.g., succinil dehydrogenase mutation/dysregulation, insuline growth factor expression, and mutations in the RAS-RAF-MAPK pathway). The classification of disease must nowadays rely on the integration of the clinico-morphological characteristics with the molecular data.

## Introduction

**Figure 1:**
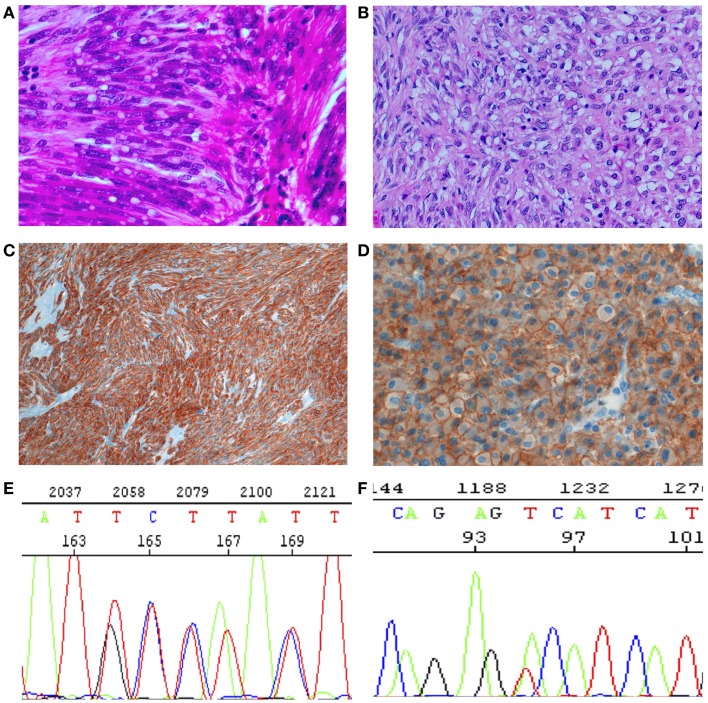
**Histology, immunohistochemistry, and molecular genetics of GISTs**. **(A,B)** Histology. **(A)** Spindle cell tumor, with paranuclear vacuoles (so-called “leiomyoblastoma”). HE 40×. **(B)** Epitheloid tumor. Large, clear cytoplasm with central nucleus. HE 40×. **(C)** Spindle cells diffusely positive for CKIT (CD117). IHC 10×. **(D)** Epitheloid cells strongly and diffusely positive for DOG1, with evident membrane enhancement. IHC 40×. **(E)** Sanger sequencing with a duplication of GCC TAT in positions 502–503 (p.A502-Y503 dup). Mutation associated with sensitivity to imatinib. **(F)**. Sanger sequencing with a substitution **(A–C)** in position 842, (p.D842V), imatinib resistant.

Gastrointestinal stromal tumors (GISTs) are the most frequent mesenchymal tumors of the gastrointestinal tract, with an incidence between 15 and 20 new cases/10,00,000/year (1, 2). The actual incidence may be, however, higher, as incidental GISTs are relatively frequent (3–6). The most frequent localization is the stomach, followed by the small intestine, the colon–rectum, and the esophagus (7). The existence of true extragastrointestinal GISTs is controversial. Although the concept that mesenchymal tumors of the gastrointestinal tract with leiomyomatous morphology are “bizarre” or “blastomatous” had been established almost 70 years ago by Stout (8, 9), the actual histogenesis of these tumors was defined about 30 years ago (in the pre-immunohistochemistry era) by Mazur and Clark (10), who proposed the non-committal term of “Gastrointestinal stromal tumors.” With the development of immunohistochemistry, the concept could be better specified. Most cases (70%) were positive for CD34, and the expression of smooth muscle markers was seen in less than 50%. CD34 is normally expressed in endothelia, in hematopoietic stem cells, perivascular fibroblasts, and stromal fibroblasts in various localization, thus underlining the “stromal,” but uncommittal nature of these tumors (11). Moreover, CD34 is expressed in a proportion of interstitial cells of Cajal (ICC), the pacemaker cells of the gastrointestinal tract (12). In 1998, two groups (13, 14) showed independently that more than 80% of these enigmatic tumors harbor constitutively activating mutations of the *ckit* gene that encode an important receptor tyrosine kinase (RTK) type III. CKIT (also known as CD117) immunohistochemical expression was contemporarily reported in more than 95% of GIST cases, thus becoming an important tool for the diagnosis (15, 16) (Figure 1). The immunophenotype is shared by the ICC (17), so that Kindblom et al. proposed in their paper the term “GIPACT,” GastroIntestinal PAcemaker Cells Tumor. This term was, however, not retained. Although sometimes questioned, the theory of origin from ICC is nowadays generally accepted. In 2003, it was shown that a substantial fraction of CKIT-wild-type GISTs’ harbor-activating mutations in *pdfgra* (platelet-derived growth factor alpha) gene, coding for another important RTK type III (18). This confirms that the oncogenesis of GISTs is probably related to early activation of RTKs. Interestingly, the immunohistochemical positivity for CD117 is independent from the mutational status of RTK genes (19). Another almost pathognomonic IHC marker is DOG-1, corresponding to the potassium transporter ANO1 (Figure 1D). The importance of the RTK mutational status is also underlined by the fact that CKIT and PDGFRA are very good target for the targeted therapy with the RTK inhibitor imatinib mesylate (Gleevec^®^, Novartis Pharma AG, Basel, Switzerland). Imatinib is now the first-line option in the medical treatment of GISTs (1, 20). Imatinib is approved in US and in Europe for adjuvant therapy (21) and may be used also in a neoadjuvant setting to reduce the tumor mass (1). Imatinib is very effective on sensitive GISTs as it was shown in the communication of Joensuu published in “The New England Journal of Medicine” in 2003 (22).

The therapy with imatinib has become the paradigm of targeted therapy in solid tumors. In spite of this great success, primary and secondary resistance to targeted therapy remains a problem to solve. Beneath a minority of GISTs that simply do not respond to the therapy with imatinib (primary resistance), due probably to their genetic constitution (see below), half of the patients develop disease progression after 2 years of treatment with imatinib (23). The main predictor of the response to therapy is represented by the mutation in the RTK genes (7, 21, 24). The genetic alterations in the RTK genes are important early events in the oncogenesis of GISTs, and define the response to targeted therapy.

Recently, mutations in BRAF and KRAS, both belonging to the RAS-RAF-MAPK pathway (25, 26), and hyperexpression of the transcription factor ETV1 (27) have been described.

The understanding of GISTs biology has stimulated the development of RTK inhibitors. Nowadays, there are at least three molecules that can be used against KIT and/or PDGFRA. The evolution of the idea of GISTs represents also how our paradigm of classification of disease is changing. From a “morphologic/etiologic” classification, we are going to more dynamic and flexible criteria, where the “old” clinicopathologic parameters are integrated with/substituted by the molecular alterations. This process is advancing also in other fields of oncology, as shown e.g., by the fourth edition of the WHO/IARC “blue books” (http://www.iarc.fr/en/research-groups/sec4/).

This review will, therefore, focus on the biology and molecular pathology of GISTs, and on the evolution of their classification.

## Molecular Alterations in GISTs

### RTK III

**Figure 2:**
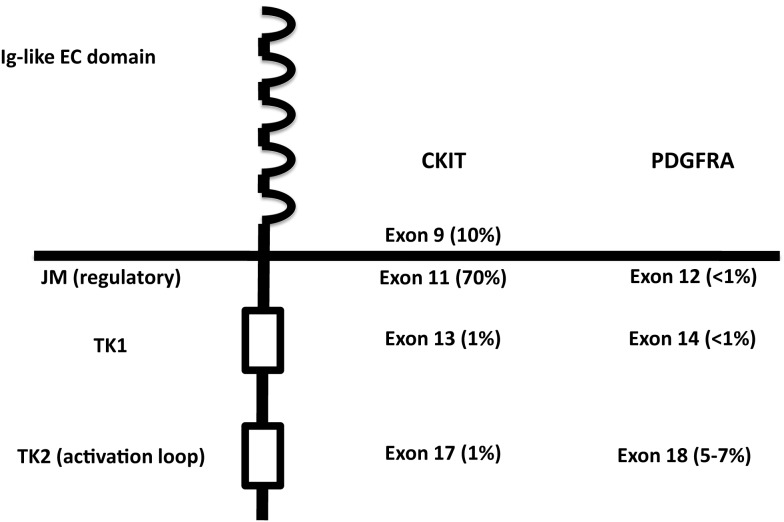
**Structure of RTK III, with localization of the activating mutations in KIT and PDGFRA**. EC, extracellular; JM, juxtamembrane; TK, tyrosine kinase.

CKIT and PDGFRA are RTK III, together with PDGFRB, macrophage colony-stimulating-factor receptor (CSFR1), FLT1, Flk/KDR, and Fl cytokine receptor (FLT3) (28, 29). RTK III have five Ig-like extracellular domains, one transmembrane domain, one intracellular juxtamembrane regulatory domain, and two intracellular tyrosine kinase domain with autophosphorylating capacity (29) (Figure 2). CKIT and PDGFRA are located on the same chromosomal region (4q12) and are very similar, both in the sequence and in the structure (29). The ligands (stem cell factor, SCF for CKIT and platelet-derived growth factor, PDGF for PDGFRA) cause homodymerization of the receptor. Subsequently, the TK domains autophosphorylate and activate, triggering the metabolic pathways of RAS-RAF-MAPK, PI3K-AKT, and signal transducer and activator of transcription 3 (STAT3) (30–34).

### CKIT

CKIT is crucial in the development of different cell types, in particular melanocytes, hematopoietic progenitor cells, mast cells, primordial germ cells, and, last but not least ICC, the probable cells of origin of GISTs (17, 21, 35). KIT-activating mutations are important for the genesis and development of several human tumor types: seminoma/dysgerminoma, mastocytosis, acute myeloid leukemia, melanoma, and GIST (36–39). CKIT can be, therefore, considered as an important oncogenetic factor in various different tissues.

Germline KIT mutations are very rare and they are associated with familial GISTs (21), while activating mutations are generally somatic and cause homodymerization of KIT and subsequent tyrosine kinase activation without SCF binding. Activating mutations are most frequently (60–70% of cases) found in exon 11 of CKIT gene (7), corresponding to the juxtamembrane intracellular regulatory domain of the protein (Figure 2). Because of exon 11 mutations, the protein changes its structure from the inactive into the active state (40). There are many different mutation types (in-frame deletions, insertions, substitutions) in various combinations (24, 29, 41). The kind of the alteration is clearly associated with clinicopathologic characteristics, such as prognosis and localization. For instance, deletions, of codons 557–558, are associated with shorter overall and disease-free survival (42–45), whereas tandem internal duplications (Figure 1E) are associated with a relatively indolent course (46, 47).

Exon 9 (corresponding to the extracellular domain of the KIT molecule) mutations are present in almost 10% of GIST cases (7, 48) and are almost only duplications of six nucleotides, corresponding to the A502_Y503 residues of the protein (49). The mutations probably induce conformational changes similar to SCF binding, leading to autoactivation. Exon 9-mutated GISTs are more often localized in small intestine or colon and have poorer prognosis (47–53). They have also different gene expression signatures (50).

Mutations in tyrosine kinase domain (exon 17) and ATP-binding site (exon 13) are rare and generally not primary (7, 34, 42, 51–60). They are generally secondary mutations arising during the targeted treatment with receptor tyrosine kinase inhibitors (RTKI), and induce secondary resistance (58–60) (see below). Most KIT exon 13 mutations are single substitutions leading to K642E in the aminoacidic sequence (52).

Recently, deletion of codon 419 in exon 8 of KIT (corresponding to the extracellular domain) have been described in a very small subset of GIST (≈0.2% of all cases) (61). This mutation was found also in systemic mastocytosis. Interestingly, it seems that this mutation is associated with sensitivity to RTKI.

Activating mutations of KIT trigger pathways, such as MAPK, PI3K-AKT, and STAT3 (30, 33, 62–66). The MAPK pathway is functionally related to several transcription factors (e.g., MYC, ELK, and CREB) and finally positively controls the cell cycle (67). The PI3K pathway, through phosphorylation of AKT and PDK1, acts against the apoptosis and indirectly stimulates the cell cycle (68). Phosphorylation of STAT3 triggers its transport in the nucleus and behaviors as a transcription factor, with a positive effect on proliferation and a negative effect on apoptosis (69).

Activation of RTK is surely pivotal in the oncogenetic pathway of GISTs, and represents an early event, but is not the exclusive factor for the acquisition of the transformed phenotype. For instance, a very interesting and elegant study has been published (27), suggesting that ETV1 (ETS translocation variant 1) may interact with KIT in the oncogenesis. The clinical importance of ETV1, however, is controversial (70, 71).

Proteasome degradation regulates physiological levels of KIT. Mutated KIT is degraded more slowly than wild-type KIT, probably because of physical interaction with heat-shock protein 90 (72, 73). HSP90 inhibitors are indeed effective in experimental models of GISTs (74).

### PDGFRA

PDGFRA has similar sequence and function as KIT. It is mutated in GISTs and AML and translocated on FILP1 in hematologic malignancies (21). Activating mutations in KIT and PDGFRA are mutually exclusive in GISTs (18, 31). Mutations in PDGFRA are in exons 12, 14, and 18, corresponding to the juxtamembrane regulatory domain and the tyrosine kinase domain of the protein, respectively (exons 11, 13, and 17 of KIT). PDGFRA-mutated GISTs are 10–12% of all cases (53, 56, 57, 75–79). They have epitheloid morphology and are generally gastric tumors, showing an indolent course (80–82). They also have different gene expression signatures (32, 83). On the other hand, the immunohistochemical phenotype (positivity for CD117, DOG-1, and PKC-Theta), the chromosomal alteration (deletion of 14q and 22q), and the biochemical properties (activated pathways and stabilization by HSP90) of PDGFRA-mutated GISTs overlap with CKIT-mutated tumors (84–87). The most frequent mutations in PDGFRA involve the aspartic acid in position 842 (Figure 1F) and are generally not sensitive to imatinib (18, 79, 88–95). Insertions and duplications are very rare, while deletions and deletions/insertions between codon 840 and 849 are more frequent. In exon 12, the most frequent mutation is a substitution T - > A, leading to a Val561Asp (19). In-frame deletions are also relatively frequent in exon 12, clustering in codons 559–72. Mutations in exon 14 are exceptional.

### RAS-RAF-MAPK pathway

In all, 10–15% of GISTs are wild type both for KIT and PDGFRA. This genotype is not related to the immunophenotype, namely with the positivity to KIT (CD117) and DOG1 stainings. Several “wt”-GISTs are strongly positive for CKIT (CD117), and the involvement of RTK has been proved functionally (phosphorylation and subsequent activation) (30). These so-called “wild-type” GISTs are a very heterogeneous group, showing different, probably oncogenic mutations. BRAF V600E has been described in 13% of “wild-type” GISTs (25, 58, 96). At the beginning, it was thought that the BRAF mutations were characteristic of “wild-type” GISTs. In a recent study, Miranda et al. (26) have suggested that BRAF is mutated in ca., 2% of GISTs carrying KIT or PDGFRA mutation, thus suggesting a further mechanism of primary resistance to imatinib treatment (see below). In the same study, 5% of GISTs carrying mutations in KIT or PDGFRA showed mutation in codon 2 of KRAS (G12A or G13D). Also, mutations in HRAS and NRAS have been found, but they are very rare. The presence and, above all, the meaning of mutations in KRAS were questioned by a recent study on 450 GISTs sequenced with Sanger’s method (97).

### Neurofibromatosis 1

Neurofibromatosis 1 (NF1) (von Recklinghausen disease) is an autosomal dominant inherited disease, characterized by multiple neurofibromas, café-au-lait spots, and other mesenchymal tumors. NF1 is caused by inactivating mutations in the gene NF1, localized on chromosome 17, coding for neurofibromin (98). Patients with NF1 have an increased risk to develop multiple GISTs, with a spindle cell morphology and with predominant intestinal location (99). The tumors have rarely, “uncommon” mutations in RTK, but CKIT and PDGFRA most often are not mutated (100–104). Neurofibromin is analog to guanosine triphosphatase (GTPase) activating proteins (GAPs), which control the level and activity of RAS. Loss of NF1 leads to high levels of active RAS. Hyperactivation of the MAPK pathway is a consequence of somatic inactivation of wild-type NF1 allele in GISTs (105). From the cytogenetic point of view, they share deletion of 14q and 22q with classical GISTs.

### Succinate dehydrogenase complex

Succinate dehydrogenase complex (SDH) is a heterotetramer composed by four subunits (SDHA-D), localized in the inner mitochondrial membrane. Subunit A oxidizes succinate to fumarate in the Krebs’ cycle. Subunit B participates in the electron transport chain for the oxidation of ubiquinone to ubiquinol, and subunits C and D (SDHC and SDHD) are membrane-anchoring subunits (106). SDH deficiency characterizes subsets of different tumors (e.g., GISTs, paragangliomas, renal cell carcinomas, and pituitary adenomas) (107, 108). SDH-deficient GISTs (identified by immunohistochemical negativity for SDHB) are the largest subgroup of “wt-GIST” (109, 110). They are always found in the stomach, are epitheloid, and often multiple and resistant to imatinib. Moreover, in contrast to “classical” GISTs, they metastasize to lymph nodes, and show activation of insuline growth factor receptor (IGFR). Their prognosis is not determined only by size, site, and mitotic index (110–116). They have an indolent course and even with liver metastases, the patients live long. SDH-deficient GISTs are the majority of pediatric GISTs in the stomach (110) and are part of two syndromes: the Carney triad (association of paranganglioma, pulmonary chondroma, and gastric GIST) and the Carney-Stratakis syndrome (association of GISTs and paragangliomas) (113, 117). The genetic events involved in the tumorigenesis of these tumors are not yet completely clarified (118, 119) and, in half of them, no mutation has been identified, although the IHC staining for SDH is negative (120). Defects of SDHx (independent from the involved subunit) induce accumulation of succinate, which inhibits degradation of HIF, subsequent increase of HIF levels, and its translocation in the nucleus, where HIF triggers the transcription of VEGF and IGF1R (121, 122), with aberrant proliferation and tumorigenic responses. A review of more than 1000 GISTs has shown that IGF1R expression is preferentially expressed in gastric SDH-deficient GISTs, but never in intestinal GISTs (123). The role of the axis IGF-IGF1R in maintaining the proliferative activity in GISTs has also been confirmed, both experimentally and on a series of SDH-deficient GISTs (124, 125).

### Chromosomal alterations

Chromosomal losses are much more frequent than chromosomal gains in GISTs. Losses of 14q, 22q, and 1p are the most frequent losses (126–128) and may subclassify GISTs in subsets with specific characteristics (129). Other losses are −9p, −11p, −17p, −10q, −13q, and −15q. Combining array CGH and transcriptome analysis Ylpaa et al. (130) have shown that the accumulation of cytogenetic changes (chromosomal losses) parallels the evolution of the tumor and could be seen as “genetic staging.” Specific genes (e.g., OXA1L on 14q and AKAP13 on 15q) are differentially expressed in the different “genetic stages.”

Cytogenetic gains are relatively rare and localized, but are associated with malignancy. In particular, gains on the loci for CCND1 and MDM2 gains have been shown to be associated with malignancy (131). On the other hand, genes involved in cell cycle control are dysregulated in high-risk tumors. Deletion or epigenetic inactivation of the gene CDKN2A, encoding for p16 and p14, two regulators of the cell cycle, is associated with malignant behavior (132–134). p16 downregulation may cause through Rb phosphorylation E2F1-dependent transcription of genes essential for late G1/S phase transition (135).

## Targeted Therapy

The prognosis of high-risk/advanced GISTs has been very poor until 2000. Surgery was the exclusive therapy, and the median survival was less than 18 months (136). The introduction of imatinib mesylate in the therapy changed dramatically this situation. Imatinib was originally developed for chronic myeloid leukemia (CML) (29). It has a very strong inhibitory activity against KIT, fixing it in its inactive conformation. After the first communication of the dramatic success of the therapy in a case of advanced metastatic GIST in a 50-year-old woman (22), it was rapidly introduced in the therapy of metastatic non-resectable tumors and is now approved also in the adjuvant setting, with a clear-cut improving of the median survival (5 years). The most important predictive factor of the response to targeted therapy is the mutational status of the RTK genes (19, 21, 97). The best response rate is achieved with mutations in exon 11 of CKIT gene that are generally associated with a high response rate (≈80%), whereas mutations in exon 9 are associated with a response rate of ≈45% (91, 137) and deserve a higher doses of RTKI (Table 1). Mutations in exon 13 and exon 17 of CKIT are generally not responsive, such as mutations in codon 842 of PDFGRA and wt-GISTs (see below).

**Table 1:** **Integration between clinicopathologic and molecular criteria in the classification of GISTs**.

Genetic alteration	Mutation	Frequency	Localization	Histology	Prognosis	Imatinib resp.	Syndromes	Remarks
**KIT-MUTATED (≈70%)**								
Exon 9 (EC)	Insertion AY502–503	≈10%	Small bowel and colon	Spindle cell	Poorer	Partially resistant (≈45% RR)	None	Higher dose requested; sunitinib
Exon 11 (JM)	W557-K558del	≈70%	Whole GI tract	Spindle cell or epithelioid cell	Poorer in stomach	Generally responsive (≈80% RR)	Many different familial GIST syndromes	
	Deletion		
	Deletion–Insertions	
	Substitutions				Better in stomach		
	Duplications		Generally stomach	Spindle cell	Better in stomach		
Exon 13 (TK)	K642E	1%	Whole GI Tract		Poorer in stomach	Responsive		
	V654A				Poorer	Resistant		Causes secondary resistance
	T670I				Poorer	Resistant		“Gatekeeper.” Secondary resistance
Exon 17 (activation loop)	Substitutions (D816, D820, N822)	0.5–1%	Whole GI tract		No prognostic value	Resistant		Secondary resistance
**PDGFRA-MUTATED (≈7%)**								
Exon 12 (JM)	Deletions/substitutions (e.g., V561D)	≈1%	Stomach	Epithelioid or mixed spindle cell/epithelioid	Indolent course	Responsive	Familial GIST syndromes	
Exon 14 (TK domain)	N659K, N659I	<1%				Responsive	None	
Exon 18 (activation loop)	D842V, D842Y	≈5%				Resistant	Familial GISTs	
	Other substitutions	<1%				Responsive	None	
	Deletions: I843, I843-H845, D842-H845, D842-M844	1%	All GI tract			Responsive	Familial GISTs
**KIT WT/PDGFRAWT (≈15%)**								
	SDHx mutation	≈2%	Stomach	Epithelioid, multifocal	Indolent course. NB: prognosis not dependent from size/mitotic index	Resistant	Carney-Stratakis syndrome (hereditary), Carney Triad (non-hereditary)	IHC always negative for SDHB, mainly female, hyperexpression of IGF1R
	BRAF V600E	≈10%	Prevalently small bowel	Spindle cell	Indolent course	Resistant	None	Possible mechanism of resistance; observed in RTK-mutated GISTs
	KRAS G12C, G13D	<1%	?	?	?	Resistant (?)	?	Reported in 5% of RTK-mutated GISTs
	NF1	<1%	Small bowel	Spindle cell	Good prognosis	Resistant	Neurofibromatosis	
	???	≈85%	All GI tract	Spindle cell or epithelioid	Indolent course	Resistant (progressive/stable disease in 69%)		?? Pathways downstream? Others?

**Figure 3:**
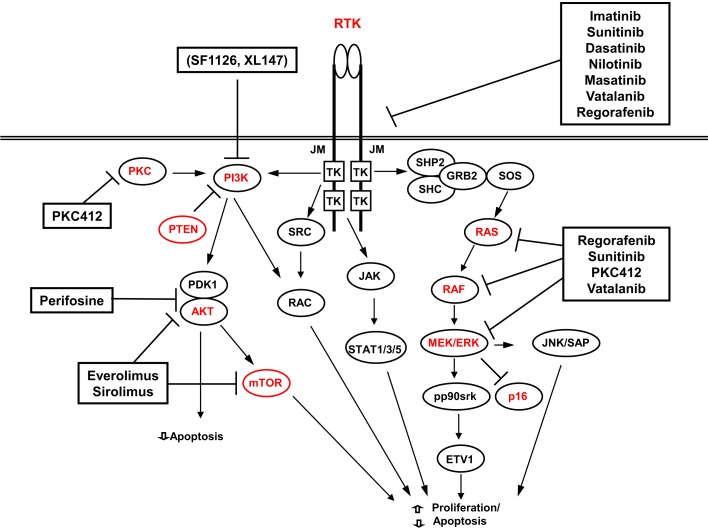
**Possible therapeutic targets (red) and targeted drugs in GISTs**.

In the attempt to achieve a better response to therapy, many other different RTKI are in use or on trial. The best results have been achieved with sunitib and regorafenib that are at present used as second- and third-line treatments (138). A summary of the possible targets and targeted drugs is shown in Figure 3.

## Resistance to Therapy

In spite of the dramatic success of the targeted therapy with RTKI, there is evidence that also long-life imatinib treatment does not destroy completely GIST cells (139, 140) and that resistance to TKI therapy, both primary and secondary, arises in most of the cases (141).

### Primary resistance

Progression within the first 6 months of treatment with imatinib means primary resistance. The only predictor of primary resistance is the mutation in RTK (CKIT and PDGFRA) genes (29, 141). The probability of primary resistance is 5% for mutations in exon 11 of KIT, 16% for mutations in exon 9 of KIT, and 23% for wild-type GIST (59, 142). Tumors with mutation in exon 9 need a higher daily dose (800 mg instead of 400 mg) (29). Other CKIT mutations (EC domain, TK domain, or activation loop, exons 8, 13, and 17) of KIT are rare and mostly associated with resistance, although in some cases a response has been reported (e.g., K642E) (61, 143). PDGFRA D842V mutation in the exon 18 is strongly resistant to imatinib therapy, while other mutations in PDGFRA are usually sensitive (91, 141, 144). The mechanisms of primary resistance in “wild-type” KIT are probably multiple and not yet clarified. One possibility is represented by alterations in molecules “downstream” RTK, such as BRAF and KRAS, as hypothesized by Miranda et al. (26). In NF1-related GISTs, that are resistant to imatinib therapy, the activation of the MAPK-ERK axis due to silencing of the NF1 gene (see above) is probably the chief factor for imatinib resistance (105). For pediatric or SDH-mutated GISTs, that are almost invariably wild type for RTK, other possible targets may be KDR (VEGFR) or mTOR (41, 109).

### Secondary resistance

As recalled above, 50% of the patients treated with imatinib relapse after 2 years (23). It is interesting to note that progression is sometimes marked by an increase in density, with or without an increase in size. A sign of progression may also be the occurrence of an area of CT-hyperdensity within a responding (hypodense) lesion. This gives rise to the so-called “nodule within the nodule” pattern (141). This underlines the need of a revision of the classical oncologic criteria for progression (145). Secondary mutations in KIT or PDGFRA genes are the cause of most of the cases of secondary resistance (23, 142, 146–148). They generally occur in the same gene of the primary mutation. In KIT, they are localized in exon 13 or 17 of KIT, corresponding to the ATP-binding pocket and to the activation loop, respectively, the most frequent being T607I in the ATP-binding pocket (exon 13), followed by V654A (exon 13) and T823D (exon 17) (60, 137, 149). In most cases, the secondary mutation in PDGFRA is D842V. It is important to note that in case of relapse constituted by multiple nodes, these are often multiclonal, with different mutations in different nodes (23, 60, 142, 150). This molecular heterogeneity can also explain the relatively low efficacy of targeted therapy against relapsing GISTs.

### Other mechanisms of resistance

Amplification of KIT or PDGFRA gene has been implicated in the development of resistance in RTK-wild-type GISTs (151). Activation of alternate oncogenic pathways is another possible resistance mechanism. This is the case of KRAS and BRAF (26) or PI3K/AKT pathway upstream of mTOR (152). IGF1R may represent another mechanism of resistance. It is expressed in subsets of RTK wild-type GISTs (58, 153). IGF1R-targeted therapy of wild-type GIST is being investigated in clinical trials (e.g., NCT01560260) (154).

These possible alternate mechanisms of resistance (primary or secondary) underline the necessity to develop schemes of therapy with a broad mechanism of action. Besides inhibitors combination and inhibitors with a broad spectrum (sorafenib, masitinib, vatalanib, nilotinib, or dasatinib that target also the VEGFR1 and VEGFR2), an important possibility is, therefore, aiming at other pathways. Promising targets are HSPs, histone deacetylases, signaling intermediates, or pathways, such as mTOR, PI3K, or MAPK [for review, see Ref. (138)].

## Conclusion

**Figure 4:**
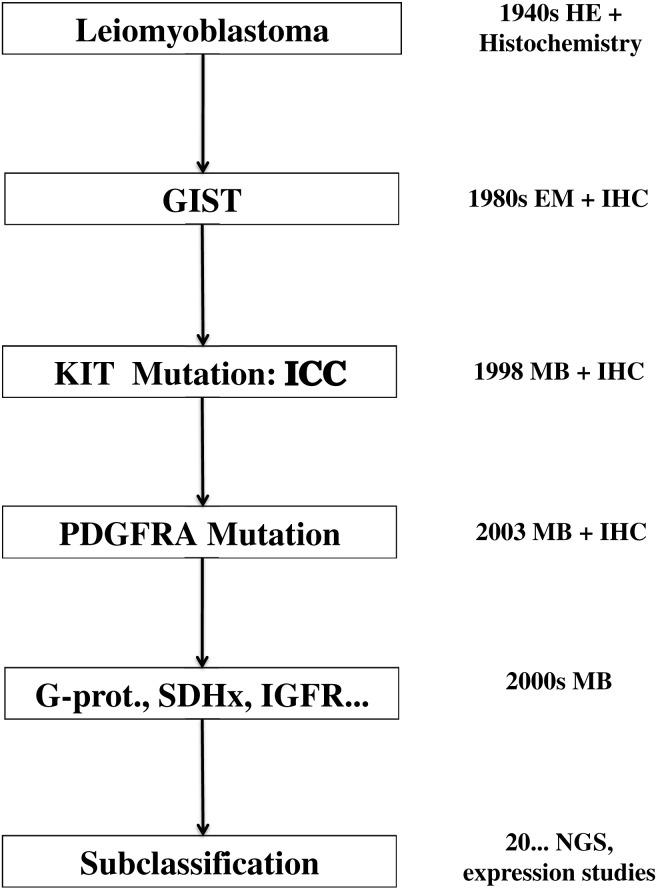
**Evolution of the concept of GISTs since 70 years**. EM, electron microscopy; IHC, immunohistochemistry; ICC, interstitial cells of Cajal; MB, molecular biology; SDH, succinil dehydrogenase; IGFR, insuline growth factor receptor; NGS, next generation sequencing.

The tremendous impact of molecular biology on modern medicine cannot be overestimated. In my opinion, the most important issue is the change of the paradigm of classification. GISTs represent a diagnostic category that changes its meaning and becomes more complex in parallel to the development of diagnostic tools and therapy (Figure 4). The non-committal term “GIST” probably, covers different “entities” (e.g., pediatric GISTs or SDH-deficient GISTs). A philosophical discussion on the actual meaning of the word “entity” in pathology and medicine goes beyond the purpose of this review. I think, however, that “entity” is an operative concept, whose content depends and is modified on the basis of the available diagnostic and therapeutic tools. This can be seen clearly in the case of GISTs. From the notion of a leiomyomatous tumor, based on HE staining, we are now dealing with a definition/classification that relies mainly on IHC stainings and molecular techniques, whereas the latter are essential for defining prognostic and therapeutic categories. The therapeutic strategy is the most important criterium for a medical classification, since the aim of medicine is the care of the patient. On the other hand, the behavior of GISTs can be defined only by integrating the molecular genetic findings with the “classical” clinicopathological parameter. A modern disease classification must, therefore, rely on the combination/integration of morphology and molecular biology (Table 1).

## Conflict of Interest Statement

The author declares that the research was conducted in the absence of any commercial or financial relationships that could be construed as a potential conflict of interest.
